# Surface Modification and Charge Injection in a Nanocomposite Of Metal Nanoparticles and Semiconductor Oxide Nanostructures

**DOI:** 10.1038/s41598-020-58308-9

**Published:** 2020-03-16

**Authors:** Bo Xiao, Gugu N. Rutherford, Amrit P. Sharma, Sangram K. Pradhan, Carl E. Bonner, Messaoud J. Bahoura

**Affiliations:** Center for Materials Research, Norfolk State University, Norfolk, VA 23504 US

**Keywords:** Nanoscale materials, Materials for optics, Nanoscale materials

## Abstract

Combining two materials in a nanoscale level can create a composite with new functionalities and improvements in their physical and chemical properties. Here we present a high-throughput approach to produce a nanocomposite consisting of metal nanoparticles and semiconductor oxide nanostructures. Volmer-Weber growth, though unfavorable for thin films, promotes nucleation of dense and isolated metal nanoparticles on crystalline oxide nanostructures, resulting in new material properties. We demonstrate such a growth of Au nanoparticles on SnO_2_ nanostructures and a remarkable sensitivity of the nanocomposite for detecting traces of analytes in surface enhanced Raman spectroscopy. Au nanoparticles with tunable size enable us to modify surface wettability and convert hydrophilic oxide surfaces into super-hydrophobic with contact angles over 150°. We also find that charge injection through electron beam exposure shows the same effect as photo-induced charge separation, providing an extra Raman enhancement up to an order of magnitude.

## Introduction

Metal nanoparticles or nanostructures can interact with the electromagnetic field at optical frequencies. A unique physical property in these nanoparticles is the strong field enhancement associated with localized plasmon excitation, which inspires development of novel devices in applications such as energy harvesting, chemical, and biological sensing. Among them, surface enhanced Raman spectroscopy (SERS) is an analytical technique with high sensitivity that enables the detection of chemical or biological analytes in trace amount far below the limit of the conventional Raman spectroscopy. The enhancement of electromagnetic fields amplifies Raman scattering signals of analytes adsorbed on rough metal surfaces, especially on the rough surfaces generated by noble metal nanostructures. The excitation of localized surface plasmon resonances (LSPRs) in the noble metals is generally considered as the main mechanism of SERS. Theoretical calculations revealed that the electromagnetic enhancement factor can be up to ~10^10^–10^12^ ^1^, reaching the level high enough for single-molecule detection. Therefore, SERS can significantly improve the sensitivity of the conventional Raman spectrometers and provides an accessible and flexible tool to emerging portable and mobile demands in applications such as medical diagnostics, environmental monitoring, food safety, national security, and rapid screening.

Noble metal nanoparticles typically exhibit SERS enhancement at sharp edges or gaps between metallic protrusions, called hot spots. Hot spots concentrate electromagnetic radiation energy within small areas, which account for the majority of the Raman scattering signals from SERS. Because the near-field behavior dominates the concentrated electromagnetic radiation in the hot spots, the field strength, as well as associated SERS enhancement, decreases rapidly within the distance of a few nanometers. Hot spots between the nanostructure gaps should be sufficiently small^2–4^. And high-density hot spots are desired to ensure consistent detection across the surface of a SERS substrate. In addition, approaches based on superhydrophobic surfaces^5,6^ and chemical enhancement^7,8^ have been pursued to push the limit of the SERS sensitivity. Covering a premade nanostructure template with a noble metal is a common method to produce SERS substrates^9–11^. The resulted size and roughness of the noble metal are substantially determined by the morphology of the template. Hence, the method lacks the ability to control the structural properties of the noble metal sufficiently. High density noble metal nanostructures also pose stringent requirements in the nanofabrication.

In this paper, we present a high-throughput method to produce high-density metallic nanoparticles on crystalline oxide semiconductor nanostructures. Our approach involves two simple deposition processes developed to independently synthesize tin oxide (SnO_2_) nanostructures and grow Au nanoparticles (AuNPs) through physical vapor deposition. We exploit the unique crystal shapes and smooth facets of SnO_2_ nanostructures to promote Au nucleation in the Volmer-Weber growth mode and create three-dimensionally distributed nanoparticles on the SnO_2_ surfaces. Moreover, our process creates a nanocomposite combining metal and oxide in the nanoscale level that has its unique material properties beyond a simple addition of the original material systems. The nanocomposite not only modifies surface wettability but also establish a heterostructure system with an extra enhancement of the SERS effect through charge separation between the metal and oxide.

## Results and Discussion

**Figure 1:**
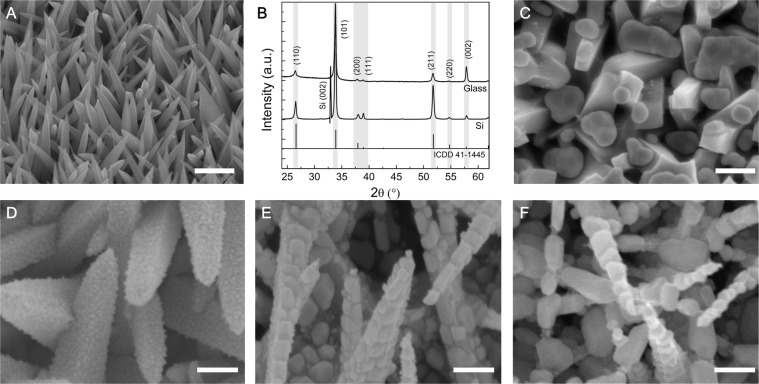
SEM images and XRD diffraction patterns. (**A**) As-grown crystalline SnO_2_ nanostructures. (**B**) XRD 2θ diffraction patterns of the SnO_2_ nanostructures on glass and (001) Si substrates. (**C**) 20 nm AuNPs grown at room temperature. (**D**) 5 nm AuNPs grown at 300 °C. (**E**) 10 nm AuNPs grown at 200 °C. (**F**) 20 nm AuNPs grown at 200 °C.

Tin oxide is a versatile optical and electrical material that has a broad range of applications in sensing, energy storage, and harvesting applications^12–19^. There are various methods to synthesize SnO_2_ nanostructures^13,20–24^. Among them, chemical vapor deposition (CVD) offers many options for customizing precursors. We have developed a single-cell CVD method based on a vapor-solid growth mechanism for large-scale synthesis of SnO_2_ nanostructures (See the details in the Supporting Information). The synthesis method produces an obelisk-like crystal nanostructure that is four-sided with a tapering sharp tip as shown in Fig. 1A. High-resolution X-ray diffraction (HRXRD) scans examined the structural properties of as-grown SnO_2_ nanostructures on the silicon and soda-lime glass substrates. Figure 1B presents the 2θ diffraction scanning patterns that reveal the primary peaks of the (110), (101) and (211) orientations, corresponding to the 2θ angles of 26.65°, 33.96°, and 51.84°, respectively. The positions of the peaks closely match the ones in the tetragonal SnO_2_ with P42/mnm space symmetry group and lattice parameters of *a* = 4.7382 Å and *c* = 3.1871 Å (ICCD card no. 041–1445). The peak intensity and sharpness indicate high crystallinity of the SnO_2_ structures and the preferable (101) orientations. Although the size, length, and density of SnO_2_ nanostructures may vary depending on the composition ratio of the precursors (Fig. S1), as-grown SnO_2_ nanostructures share similar crystallinity and diffraction patterns on glass and Si substrates. All show the dominant (101) orientations and the peak intensity ratios of I_101_/I_110_ and I_101_/I_211_ are more than 2.

We use a physical vapor deposition (PVD) technique, electron beam evaporation, to grom AuNPs on the facets of crystalline SnO_2_ nanostructures. The process involves heterogeneous growth and nucleation of metal vapor in a condensed phase. It is entirely different from coating nanostructures with an evaporated metal where the nanostructures act as a supporting material to introduce nanoscale roughness for the metal. In contrast, the growth process seeks to form AuNPs with their distinct morphology that is independent on the structural feature of the SnO_2_. Although the PVD is one of the popular techniques for depositing metals, the growth of metal nanoparticles requires more precise control of the deposition condition such as deposition rate and substrate temperature.

We adjusted such growth parameters to maintain the metal deposition in Volmer-Weber or three-dimensional island growth mode. Figs. 1C–F present scanning electron microscopy (SEM) images to reveal the morphological evolution of AuNPs at a varying substrate temperature *T*_*s*_ and deposition rate *D*. Deposited at the room temperature of *T*_*S*_, most of the Au particles aggregate in the valleys of SnO_2_ nanostructures and their sizes vary in a wide range from 20 nm to 200 nm (Fig. 1C). It is a common issue because of the poor adhesion of Au and the low surface energy of the crystalline oxides. To improve the adhesion, we heated the substrate to increase the surface diffusion and slow the solidification rate of impinging particles, allowing Au to make better contact with the SnO_2_ surfaces.

On the other hand, impinging Au atoms have a long mean free path with a line-of-sight impingement onto the substrates. Heating the substrates helps the atomic surface diffusion of Au atoms and reduces the shadowing effect. Therefore, Au atoms can absorb, diffuse, and grow on the facets of individual crystalline SnO_2_ nanostructure. The improved adhesion was observed as the *T*_*s*_ was over 100 °C. The nanoparticle growth can be considered as a particular growth mode (island or Volmer-Weber) of thin film deposition. To avoid a layer-by-layer growth or aggregation, we calibrated the growth parameters and found that the optimal condition to form dense and isolated AuNPs were at the substrate temperate in range of 150–300 °C with the deposition rate of 0.5 nm/s. Figure 1D shows the improved surface coverage of AuNPs that were grown at 300 °C of *T*_*S*_. The growth lasted 100 seconds at a deposition rate of 0.5 Å/s, equivalent to 5 nm film and creating the nanoparticles in the size of 10~20 nm. At the same deposition rate, we can adjust the growth time to control the nanoparticle size. As for the equivalent 10 nm and 20 nm growth, larger particles in the ranges of 30–60 nm and 50–120 nm were formed on the facets of the SnO_2_ (Fig. 1E,F). The growth method enables simple control of the nanoparticle size and the formation of high-density AuNPs.

**Figure 2:**
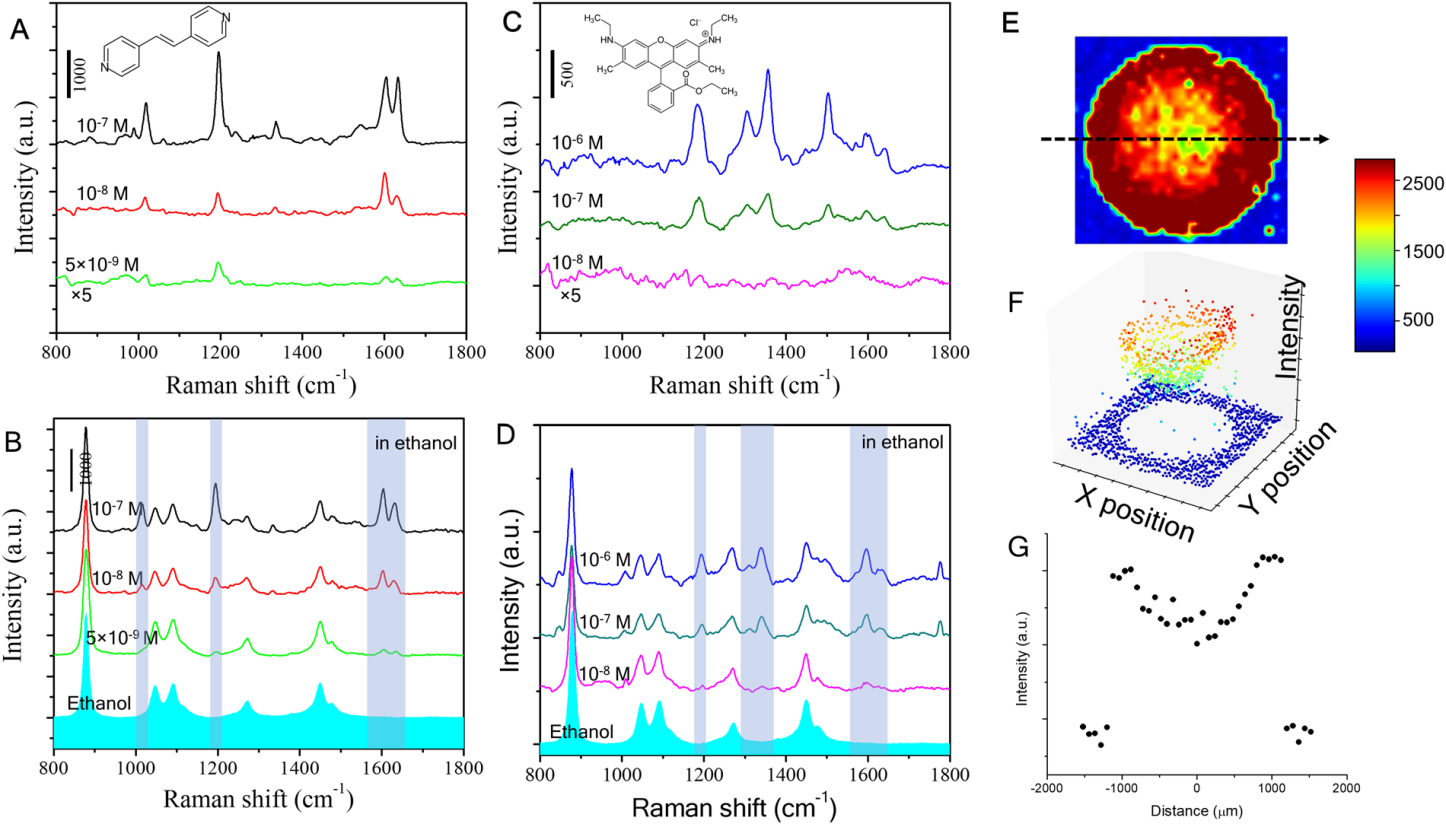
Contact angle measurements. Images of a water droplet on the surfaces of (**A**) SnO_2_ nanostructures, (**B**) Au thin film, (**C**) 20 nm Au deposited on SnO_2_ nanostructures at the room temperature, (**D**) 10 nm Au deposited on SnO_2_ nanostructures at 200 °C, (**E**) 15 nm Au deposited on SnO_2_ nanostructures at 300 °C and (**F**) 20 nm Au deposited on SnO_2_ nanostructures at 300 °C. (**G**) Illustration of the wetting absorption. (**H**) Contact angles versus time measurements during evaporation of the water droplet on the surface of (**D**). (**I**) A water droplet (15 μL) suspended on the super-hydrophobic surface.

The uniform coverage of AuNPs on SnO_2_ nanostructures changes the surface properties macroscopically. Wettability, one of the essential surface properties is a direct measure of hydrophobicity and hydrophilicity or surface energy that is an important factor affecting functional interfaces in various chemical and biological processes that occurred on the surfaces. Wettability of nanostructures has been explored to improve or introduce functionalities such as absorbing, collecting, transferring or enriching analytes in their liquid solution for sensing applications^5,6^. Fig. 2 presents contact angle measurements to demonstrate the modification of surface wettability by means of the morphology control of AuNPs grown on SnO_2_ nanostructures. SnO_2_ or its nanostructures, in general, have hydrophilic surfaces. The hydrophilic behavior can be seen in Fig. 2A where the water droplet spreads out on the surface. The tapering tips of SnO_2_ nanostructures are not an ideal support structure for the water droplet. The surface energy of the facets plays a critical role in the wettability. Because of the dominant (101) surfaces with relatively large surface energy (surface free energy of the SnO_2_ crystal faces: (001) > (101) > (100) > (110))^25,26^, water can penetrate into the nanostructures along their (101) surfaces. Although Au thin films have a larger contact angle (~80°) (Fig. 2B), SnO_2_ deposited with Au at the ambient temperature showed no signs of the improved wettability (Fig. 2C). At an elevated substrate temperature, the formation of AuNPs and their improved coverage completely change the hydrophilic surfaces. SnO_2_ nanostructures with AuNPs grown at the substrate temperature of 200 °C shows a super-hydrophobic state with a contact angle of 152.4° (standard deviation 1.2°) as shown in Fig. 2D.

**Figure 3:**
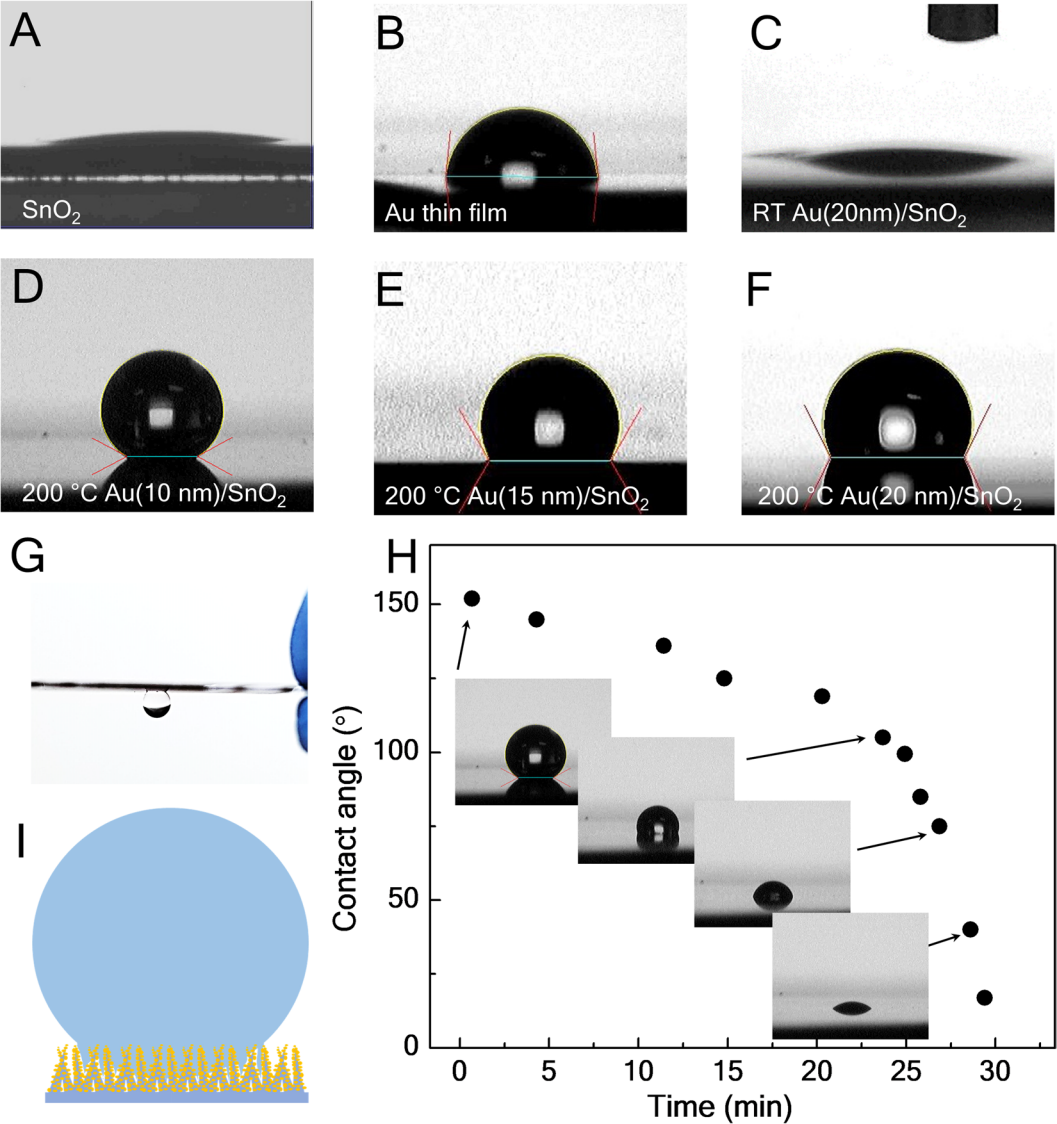
SERS measurements of BPE and R6G molecules in the ethanol and water solutions. (**A**) SERS spectra of dried BPE in ethanol, (**B**) Spectra of the SERS substrate submerged in the BPE ethanol solutions, (**C**) SERS spectra of dried R6G in ethanol, (**D**) Spectra of the SERS substrate submerged in the R6G ethanol solution, (**E**) Raman mapping scan of a drop of 10^−6^ M R6G aqueous solution, (**F**) 3D image of the peak intensity at 1180 cm^−1^. (**G**) The peak intensity of 1180 cm^−1^ along line L in (**E**).

The coverage of AuNPs reduces the exposed SnO_2_ surfaces, which lowers the surface energy and induces the transition from hydrophilicity to hydrophobicity or super-hydrophobicity. Most importantly, the gaps between AuNPs can trap air and let the water drop sit partially on the air gaps that enhances hydrophobicity as described in the Cassie model^27^. Increasing the nanoparticle size or the aggregation may reduce the trapped air and hence the contact angle. As shown in Fig. 2E,F, the contact angles dropped to 135° and 122°, corresponding to 15 nm and 20 nm Au deposition.

Furthermore, we found that the water drop could adhere to and suspended on the surface (Fig. 2G). Time dependence of the contact angle (Fig. 2H) shows that the contact angle decreased as the drop evaporated and reduced in volume. The water drop is pinned in the surface structure. It suggests that the drop penetrates the SnO_2_ nanostructures and sits on the AuNPs as illustrated in Fig. 2I. This generates an adhesion force that prevents the drop collapsing during evaporation or rolling on the surface.

An important aspect of SERS is to bring analytes close to the hot spots in trace amount detection. A major advantage of our AuNP and SnO_2_ composite is the three-dimensional distribution of the SERS active nanoparticles that increases the chance of contact between the analytes and the hot spots. In order to evaluate the potential of SERS performance, we investigated the detection using two chemicals, trans-1,2-bis-(4-pyridyl) ethylene (BPE) and rhodamine 6 G (R6G). Two methods for sample preparation were implemented in our tests: one is to drop-cast a solution-based analyte and wait for it dry before acquiring Raman spectra, and the other is to immerse a SERS substrate in a solution-based analyte during the acquisition. Nonaqueous liquid, ethanol was used to dissolve and dilute the test analytes. Because of low surface tension in ethanol, a drop of the ethanol solution spreads out and evaporates rapidly on the SERS substrates, which in practice is more desirable for simple and fast detection. In the drop-cast preparation, we applied 8 μL ethanol solutions with various analyte concentrations on the SERS substrates. In immersion preparation, we followed the evaluation method^28^ and placed the substrate in a disposable sample box (26 mm × 26 mm) with 1.5 mL of an analyte solution. Figs. 3A–D compare the Raman spectra of BPE and R6G in the two preparation methods. Raman spectra were acquired by Horiba micro-Raman system using a 785 nm laser with an accumulation time of 2 seconds. Peaks marked with gray bands in the spectra are the typical Raman scattering fingerprints of BPE and R6G. The observed Raman spectra are in good agreement with the previous reports. Our systematic measurements demonstrate the AuNPs on the oxide nanostructures can achieve the detection of analytes at concentrations down to 5 × 10^−9^ M (BPE) and 1 × 10^−8^ M (R6G). In our study, such SERS performance is very consistent in AuNPs grown at 200 °C with equivalent growth thickness about 10–20 nm. However, AuNPs grown at the ambient temperature failed to detect BPE analytes even at the concentration of 10^−5^ M due to the formation of low density and large aggregated Au particles. Furthermore, SERS performance depends on the laser wavelength and the kind of noble metal. We verified our growth method using Ag nanoparticles, which achieved similar SERS at the excitation wavelength of a 532 nm laser.

SERS efficiency for detecting low concentration analytes in aqueous solution is also improved with the superhydrophobic surface, which holds a drop of the aqueous solution onto a small area. As the solution evaporates, the accumulation enriches the analytes that contact with the high-density AuNPs. Using Raman mapping with a laser spot diameter of 2 μm and a step size of 75 μm, we collected Raman spectra across the surface area (3 × 3 mm) applied with 8 μL of 10^−7^ M R6G in water. Figs. 3E,F show color maps of the average peak intensity at 1200 cm^−1^. Although all the test points inside the analyte contact region showed strong R6G Raman spectra, the peak intensities at the edges reach an average of 30% higher than those of the center (Fig. 3G). The pattern of peak intensity along the perimeter of the evaporated drop indicates the “coffee ring” effect. The contact line of the drop was pinned on the superhydrophobic surface as the drop was evaporating.

**Figure 4:**
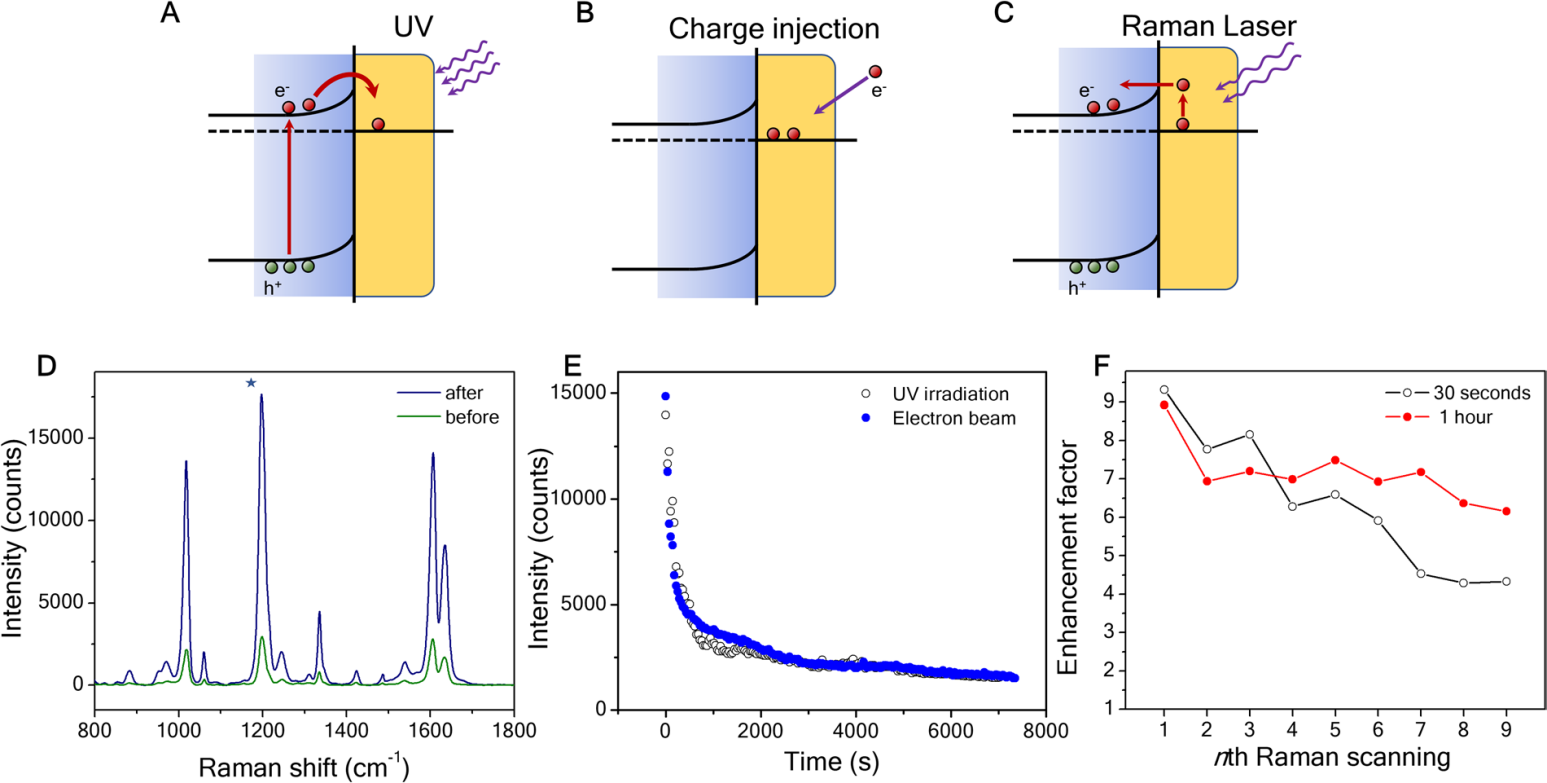
Charge injection enhanced SERS. (**A**) Illustration of charge injection by UV irradiation, (**B**) Illustration of charge injection by electron beam exposure, (**C**) Illustration of an inverse process to pump electrons from the plasmonic metal back to the oxide semiconductor, (**D**) SERS spectra before (green line) and after (blue line) the electron injection, (**E**) Raman intensity of BPE 1200 cm^−1^ peak decay vs time for the SERS substrate after the exposure of the electron beam, (**F**) Raman intensity (1200 cm^−1^) decay vs time for the SERS substrate after UV irradiation.

The composite of a plasmonic metal and an oxide semiconductor a heterostructure that enables an extra enhancement of Raman signals, because of electrons accumulated in the plasmonic metal. Photo-irradiation can induce electron accumulation, resulting in high electron density and net charges. The effect involves charge transfer and separation in the heterostructure and the extra enhancement has mainly been reported in the system of plasmonic NPs on TiO_2_^8,29,30^. Schottky contacts and semiconductor photocatalysis may well explain the mechanism of the charge behavior^31^, though the increased Raman signals are attributed to chemical enhancement from photogenerated electrons^8,29^. Electrons are excited from the valence band (VB) to conduction band (CB) in the semiconductor, and if a metal with proper work function is in contact with the semiconductor, the excited electrons can spill over from the semiconductor into the metal and then be trapped in the metal owing to the Schottky barrier (Fig. 4A). Ultraviolet (UV) light irradiation is a common method to generate electron-hole pairs from the semiconductor, but it usually takes a long time to effectively excite and inject electrons into the plasmonic metals because of the large bandgap and bulk recombination in the oxide semiconductors. Since net charges are the main factor to affect this extra enhancement, we introduce an alternative method to directly inject electrons into the plasmonic NPs (Fig. 4B) by means of a scanning electron microscope. On the other hand, there is an inverse process that will drive the electrons back to the oxide semiconductor, because the Raman laser can excite the electrons in the metal to overcome the Schottky barrier (Fig. 4C).

To verify the effects of the charge injection, we implemented two methods to compare the injections of electrons into the AuNPs through UV irradiation and electron beam injection. In the UV irradiation, the SERS substrates with the analyte were exposed under 254 nm light for 4 hours. Because the energy bandgap *E*_*g*_ of SnO_2_ is 3–4 eV^32^, the UV light with 254 nm (4.9 eV) is higher than the *E*_*g*_ of SnO_2_ but out of the range for exciting surface plasmons. The electron-hole pairs generated by the UV radiation are more likely to inject electrons from the SnO_2_ into the AuNPs. In the electron beam injection, the substrates were scanned/exposed by an electron beam from a scanning electron microscope. The substrates were placed on an insulating glass side and loaded into the microscope under the electron beam exposure (or scanning) for 5 min at 1 μA and 5 kV.

Raman measurements were carried out immediately after the exposure to minimize the charge dissipation. In the measurements, 10^−7^ M BPE was used as a standard analyte to investigate the influence before and after the charge injection process (Fig. 4D). Both charge injection methods yielded large enhancement factor, and the average intensities of Raman peaks came up to one order of magnitude higher than those of the unexposed. And we also collected Raman spectra from a repetitive scanning with a time interval of 30 s at the same spot. As shown in Fig. 4E, the peak intensity (1190 cm^−1^ BPE) drops instantly once the measurement starts. The spectra from UV irradiation and electron beam exposure show a close resemblance in the time dependence (Fig. 4E). The intensity decay indicates a reverse charge flow bringing excess electrons back to the semiconductor (Fig. 4C). The inverse process induced by the Raman laser strongly suggests the charge separation of plasmon hot carriers in the plasmonic nanostructures^30,33,34^. The net charges increase the electron density in the AuNPs. Surface plasmons excited by Raman lasers can transfer energies to the electrons and generate hot electrons with energies higher than the Schottky barrier. Thus, continuously collecting Raman spectra from the same spot accelerates the deterioration of the enhancement. But the measurement did not represent the retention rate of the net charges. We note that the enhancement of net charges lasts days after the exposure. Figure 4F shows the comparison of the Raman peak intensities from the same exposed sample at two different spots. One was collected every 30 seconds, and the other is every 1 hour. In both cases, the signals show a trend of decreasing magnitude. But for 9-time Raman acquisitions in 240 seconds, the enhancement factor dropped over 50% from 9.3 to 4.2, whereas the factor was still over 6 after 8 hours. The results imply that the net charges induced by UV or electron beam may stay a long time in the AuNPs and Raman laser gives rise to the charge dissipation. Besides, a continuous Raman acquisition in a short time generate heat on the sample surface, and the accumulated heat also accelerates the charge dissipation. Since the deterioration occurs after multiple-time acquisitions, it won’t be a practical issue to affect the Raman performance. Finally, studies are necessary to quantify the processes of the charge injection and dissipation and further investigate these effects in different pairs of plasmonic metals and oxides for applications focused on the surface characteristics or charge injection.

## Conclusion

In summary, our work demonstrates a nanocomposite consisting of high-density Au nanoparticles and crystalline SnO_2_ nanostructures. Although we focused on Au nanoparticles and SnO_2_ nanostructures in our study, the growth mechanism can be readily adapted to other similar material combinations. Because of the well-established techniques and industrialized equipment in vapor phase deposition and physical vapor deposition, our approach offers a viable way for high-throughput and large-scale fabrication. The remarkable sensitivity of SERS makes the nanocomposite a low-cost and reliable building block for SERS substrates. The growth, surface modification, and charge injection could expand numerous possibilities for new composites with different pairs of metal and oxide nanoparticles/nanostructures, which opens doors to many other applications such as high-efficiency solar cells, energy conversion, and storage devices.

## Supplementary information

Supplementary Information.
