# Publisher Correction: Surface Modification and Charge Injection in a Nanocomposite Of Metal Nanoparticles and Semiconductor Oxide Nanostructures

**DOI:** 10.1038/s41598-020-66551-3

**Published:** 2020-06-04

**Authors:** Bo Xiao, Gugu N. Rutherford, Amrit P. Sharma, Sangram K. Pradhan, Carl E. Bonner, Messaoud J. Bahoura

**Affiliations:** Center for Materials Research, Norfolk State University, Norfolk, VA 23504 US

Correction to: *Scientific Reports* 10.1038/s41598-020-58308-9, published online 16 March 2020

This Article contains an error in the order of the Figures. Figures 2 and 3 were published as Figures 3 and 2 respectively. The correct Figures 2 and 3 appear below as Figures 1 and 2. The Figure legends are correct.

**Figure 1:**
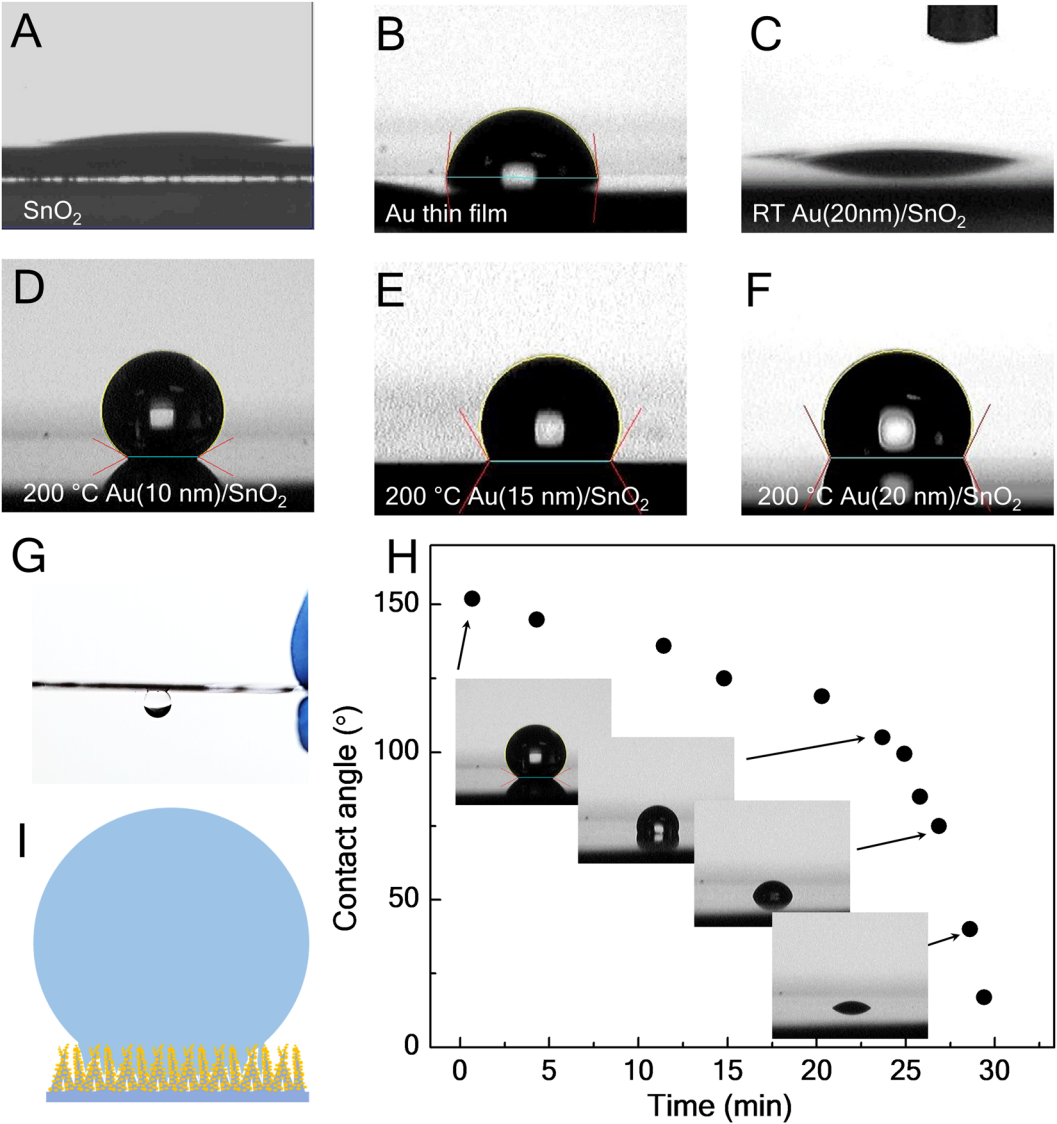
.

**Figure 2:**
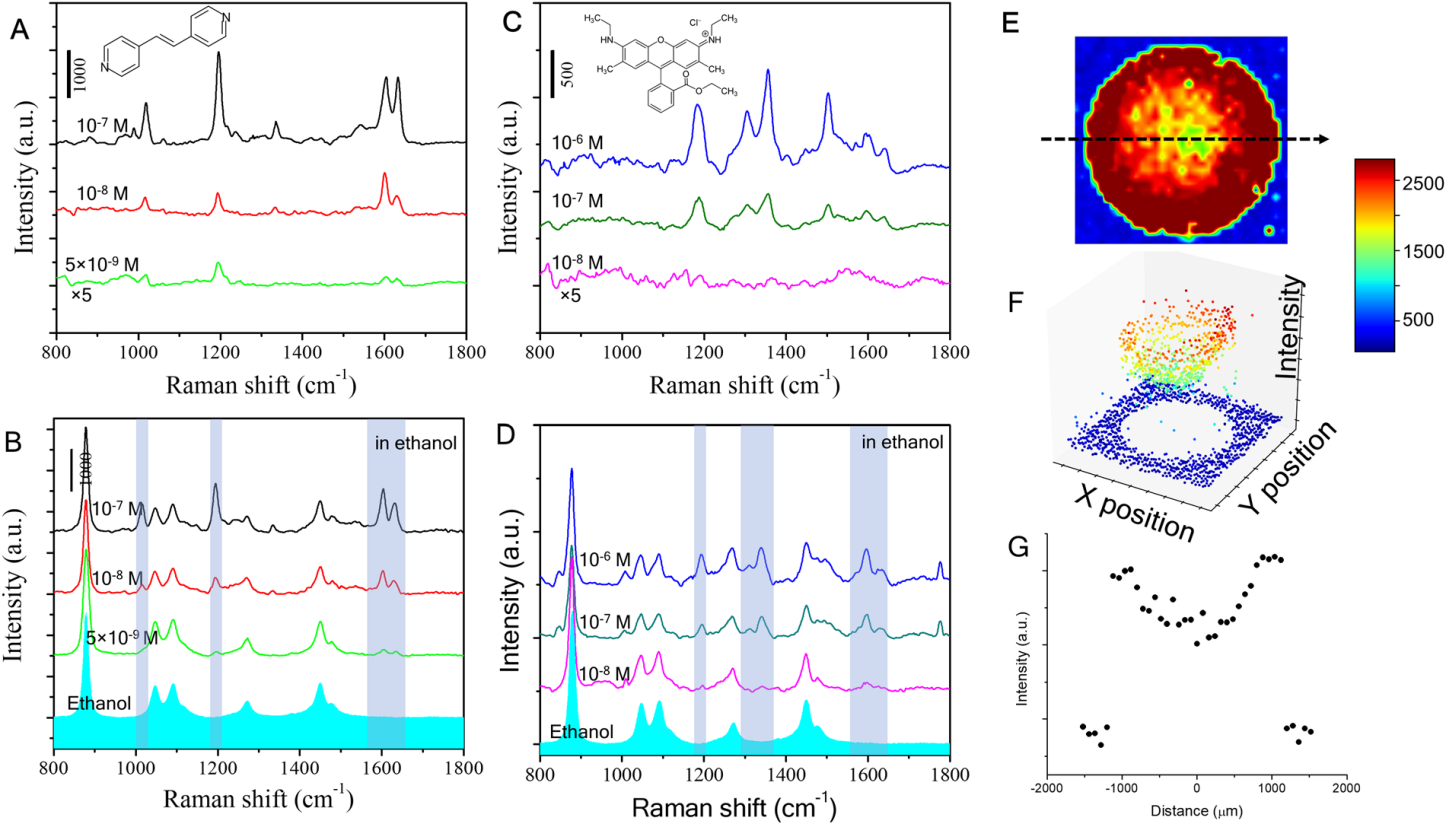
.

