# Supplementary material for: Surface Modification and Charge Injection in a Nanocomposite Of Metal Nanoparticles and Semiconductor Oxide Nanostructures
Source: Sci Rep. 2020 Mar 16;10:4743. doi: 10.1038/s41598-020-58308-9 (PMC7075916; doi:10.1038/s41598-020-58308-9)
Supplement: Supplementary file 1 — Supplementary Information. [file 41598_2020_58308_MOESM1_ESM.docx]

Supporting Information for

Surface Modification and Charge Injection in a Nanocomposite Of Metal Nanoparticles and Semiconductor Oxide Nanostructures

Bo Xiao, Gugu N Rutherford, Amrit P Sharma, Sangram K Pradhan, Carl E Bonner and Messaoud J Bahoura

*Center for Materials Research, Norfolk State University, Norfolk VA, 23504*

Large-scale SnO_2_ synthesis

Synthesis of crystalline SnO_2_ nanostructures is operated in a conventional muffle furnace that heats our custom-made cell reactor. For the growth, the reaction cell with the precursors and the substrate is transferred in the furnace at the room temperature. The furnace temperature is programmed to increase to 550 °C at a constant rate of 12 °C/min. As illustrated in Figure S1(a), the cell reactor is configured as analogous to a kitchen pot with a lid in which the substrate is placed face down to cover the cell like a lid for providing condensation and nucleation sites of the precursor vapors. Tin chloride (SnCl_2_) powder having a melting point at 247 °C is the essential precursor. The synthesis relies on evaporation to transport the precursor vapor at the atmospheric pressure. The typical reaction of the formation of SnO_2_ is:

$$\text{2SnC}\text{l}_{\text{2}}\left( \text{g} \right)\text{ + 2}\text{H}_{\text{2}}\text{O + }\text{O}_{\text{2}}\to\text{ 2SnO}\text{}_{\text{2}}\text{ + 4HCl}$$

The previous reports demonstrated that zinc chloride (ZnCl_2_) can assist the formation of SnO_2_ nanostructures.^[1,2]^ ZnCl_2_ is a highly hygroscopic material with a melting point close to SnCl_2_ at 275 °C - 290 °C. It is general in the hydrate form (ZnCl_2_·*x*H_2_O) when exposed to air, so zinc chloride hydrate supplies water for the reaction. Since SnO_2_ is difficult to dissolve in acids and alkalis but ZnO is opposite, the by-product HCl suppresses the formation of ZnO. XRD measurements showed no signs of diffraction features from chlorides and ZnO. Although the exact mechanism of the ZnCl_2_ precursor is unclear, the result implies that zinc chloride vapor acts more like an interspace separator that disturbs the lateral film growth but ameliorates the vertical growth for the formation of crystalline nanostructures. We also found that SnCl_2_ water solution (~1 M) alone can be used in the method to produce SnO_2_ nanostructures. But this precursor showed poor reproducibility and uniformity that the SnO_2_ nanostructures tend to be sparsely distributed and a mixture of micro- and nanostructures. The similar evaporation condition of ZnCl_2_ and SnCl_2_ and the coexistence of their vapors may suggest why ZnCl_2_ works well to promote the formation of uniform nanostructures.

The evolution of the nanostructure nucleation is a complicated interplay among temperature, particle flux, and atomic surface diffusion. The other advantage of using ZnCl_2_ and SnCl_2_ precursors is that the ratio of the two precursors can be easily changed to modify the nanostructure morphology. It allows us to design the size and shape for maximizing SERS performance. In our experiment, the reactor cell with the inner diameter of 75 mm and the height of 10 mm was employed for the standard 3-inch wafers. According to the volume of the reactor cell, we applied different mixtures of the precursors to study the nanostructure growth. Figure S1 (b) – (e) shows the SEM images of the nanostructure using 200, 300, 400 and 500 mg tin chloride with the same amount of 600 mg zinc chloride. The variation of the amount of tin chloride has an equivalent effect of particle flux modulation, and more tin chloride precursor results in an increased particle flux at the same heating rate. At 200 mg, whisker nanowires (Figure 1b) are the dominate structures having an average size of 20 – 50 nm and 3 μm in length. As increasing the amount of tin chloride, the structure changes to be obelisk shape with the average size of 50 -100 nm and 2 μm in length (Figure 1c). Using 400 mg tin chloride can produce highly packed nanorods, but further increasing the weight to 500 mg results in thin film nucleation with grain structures of 100 – 300 nm (Figure 1d and 1e).


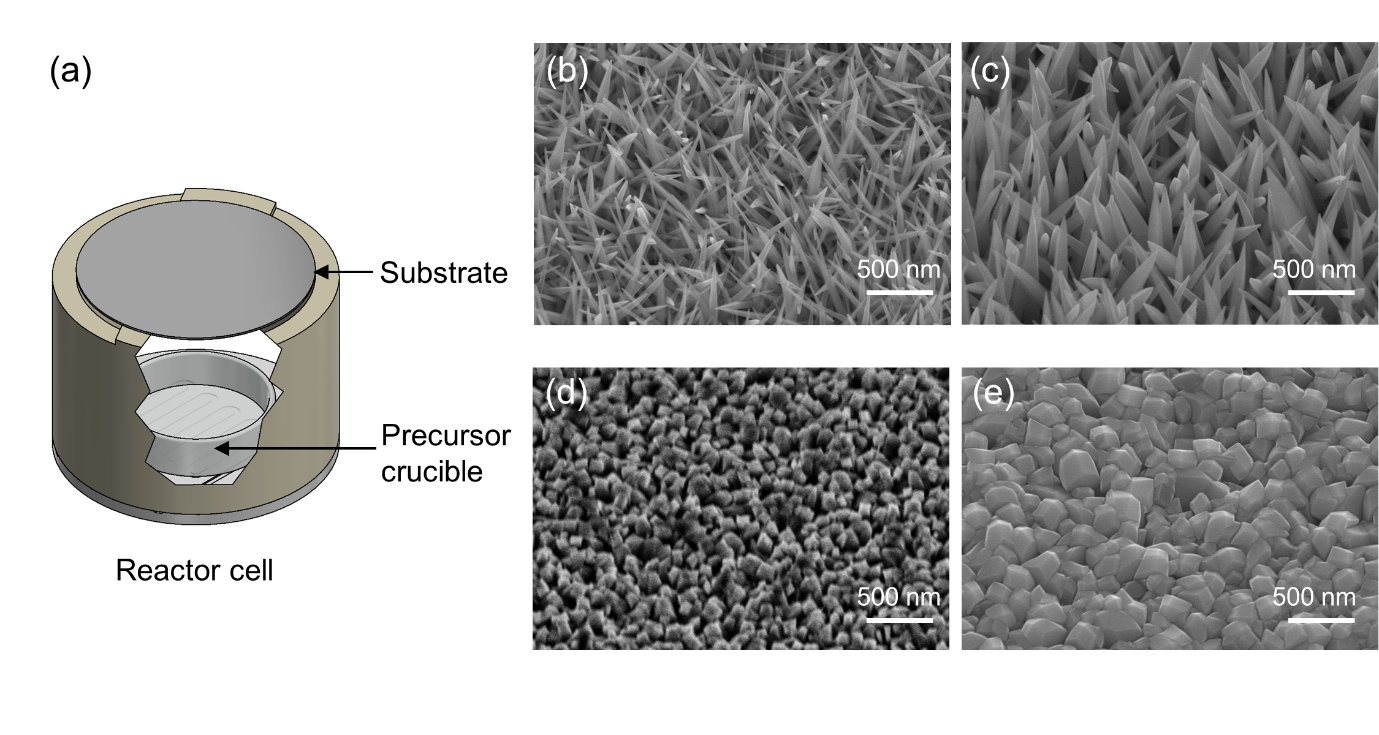


Figure S1 SnO_2_ nanostructure synthesis. (a) Diagram of the single-cell vapor phase deposition. (b) Nanowire, (c) Nano-obelisk, (d) Nanorod, and (e) Large grain thin film.

REFERENCE

[1] X. Wang, W. Liu, H. Yang, X. Li, N. Li, R. Shi, H. Zhao, J. Yu, *Acta Mater.* **2011**, *59*, 1291.

[2] C. G. Carvajal, C. Snow-Davis, R. Mundle, a. K. Pradhan, *J. Electrochem. Soc.* **2014**, *161*, B3151.
